# Acknowledgement to Reviewers of *Tropical Medicine and Infectious Disease* in 2017

**DOI:** 10.3390/tropicalmed3010007

**Published:** 2018-01-10

**Authors:** TropicalMed Editorial Office

**Affiliations:** MDPI AG, St. Alban-Anlage 66, 4052 Basel, Switzerland

Peer review is an essential part in the publication process, ensuring that *Tropical Medicine and Infectious Disease* maintains high quality standards for its published papers. In 2017, a total of 64 papers were published in the journal. Thanks to the cooperation of our reviewers, the median time to first decision was 20 days and the median time to publication was 44.5 days. The editors would like to express their sincere gratitude to the following reviewers for their time and dedication in 2017:
Alberdi, PilarLuce-Fedrow, AlisonAlgeo, Timothy P.Lyon, LizAmery, RobertMacaluso, KevinAnderson, BurtMaina, AliceAnnette, Pruss-UstunMakita, KoheiArnold, BenjaminMarques Allegretti, SilmaraAvilés, Marta MartínezMasatani, TatsunoriAzad, AbduMcBride, JohnBaird, Robert W.McIntosh, E. David G.Bajer, AnnaMehlhorn, HeinzBarbier, MarietteMessina, JaneBarichello, TatianaMinter, AmandaBasu, PratyushaMiranda, Mary Elizabeth G.Batt, Ryan D.Moon, TroyBelo, SilvanaMoore, SusanBergquist, RobertMuehlenbachs, AtisBernardini, GiuliaMuller, ThomasBernardo, UmbertoMuñoz-Antoli, CarlaBisoffi, ZenoMurdoch, Michele E.Blacksell, StuartMurphy, JuliaBoulanger, JasonMusto, JennieBowen, Richard A.Mutinelli, FrancoBowman, DwightMwangi, ThumbiBradbury, RichardNadin-Davis, Susan A.Brookes, VictoriaNeville, PeterBrown, Catherine M.Nishizono, AkiraBrown, MikeNoland, Gregory S.Brown, GrantOrtu, GiuseppinaBudge, Philip J.Padovese, ValeskaBuonfrate, DoraPaini, DeanBurrows, MalcolmPlain, Karren M.Calisher, CharlesQueiroz, Luzia HelenaCampagnolo, Enzo RiccardoRaby, EdwardCarr, JillRamaswamy, KCascio, AntonioRecuenco, SergioChami, GoyletteReesChen, Lin H.Ribeiro, Rita Cláudia CardosoChing, Wei MeiRibes, JulieCoelho, Ana CláudiaRichards, AllenCoertse, JessicaRobardet, EmmanuelleColeman, KenRobertson, AndyColubri, AndresRobinson, Derrick R.Cronin, AidanRollinson, DavidCrow, BenRöltgen, KatharinaCutler, Sally JaneRombo, LarsDavis, April D.Rosa, Bruce A.De Thoisy, BenoitRosen, SydneyDharmarajan, GuhaRoss, MaryEckerle, IsabellaRozsa, LajosEidson, MillicentRudge, JamesElahi, ShokrollahRuizElse, KathrynRupprecht, CharlesEscobar, LuisSakuntabhai, AnavajFahrion, Anna S.Sarovich, DerekFinke, StefanSarti, ElsaFlegg, JenniferScotti, MarcusFooks, TonySleigh, AdrianForsyth, Colin J.Smith, Todd G.Freuling, ConradSmith, David W.Fu, ZhenfangSnyder, LoriGaardbo Kuhn, KatrinSt. Juliana, Justin R.Gazzaniga, ValentinaStahl, Jean-PaulGeoghegan, Jemma L.Stanton, MichelleGnanadurai, ClementStephen, DimityGordon, Catherine A.Stewart, Anna M.Gottdenker, NicoleStolk, Wilma A.Gryseels, SophieStrande, LindaGunesekera, AmilaTan Soo, Jie ShengHasegawa, HideoTasmin, SairaHattaf, KhalidTaylor, LouiseHay, Roderick J.Taylor, TimHigashi, TomomiTeo, AndrewHodgson, IanTheimer, Tad C.Holmen, SigveTiittala, PaulaHostnik, PeterToledo, RafaelHribar, LawrenceTrewby, HannahHudson, IreneTruscott, JamesIde, KazukiTsioutis, ConstantinosIliev, Asparouh I.Undurraga, Eduardo A.Innocent, Gilesvan Gucht, StevenJackson, Alanvan Lieshout, LisetteJamshed, ShziaVelasco-Villa, AndrésJarvis, JodieVeraldi, StefanoJohnson, Shylo R.Viducic, DarijaJones, MalcolmVos, AdJoseph, HayleyWahlstrom, HeleneKanki, PhyllisWallace, Ryan M.Kelly, PatrickWallace, John W.Kelly, DarylWangrangsimakul, TriKobayashi, YukiWarrell, MaryKorou, Laskarina-MariaWasniewski, MarineKroidl, IngeWeir, DawnKuo, Chi-ChienWelkhoff, PhilipLamberton, PoppyWeyer, JacquelineLammie, PatrickWilloughby, Rodney ELaverack, GlennWorboys, MichaelLi, YuYakobson, Boris A.Liang, SongZienius, DainiusLontok, ErikZinnstag, JakobLow, Jenny G.


